# A Case of Endosteal Hyperostosis Caused by a Mutation of the Low-Density Lipoprotein Receptor-Related Protein 5 (LRP5) Gene

**DOI:** 10.7759/cureus.85100

**Published:** 2025-05-30

**Authors:** Lauren H Beshay

**Affiliations:** 1 Endocrinology, David Geffen School of Medicine, University of California, Los Angeles (UCLA), Los Angeles, USA

**Keywords:** autosomal dominant osteosclerosis, endosteal hyperostosis, high bone mass, mutation of lrp5 gene, torus palatinus

## Abstract

Worth syndrome, also known as autosomal dominant osteosclerosis and high bone mineral density, is a rare disease caused by a gain-of-function mutation of the low-density lipoprotein receptor-related protein 5 (LRP5) gene leading to endosteal hyperostosis. It is characterized by increased bone density and benign bony structures on the palate, known as torus palatinus. The skeleton is normal in childhood, but facial metamorphoses occur in adolescence, as the mandible becomes elongated and the forehead flattens. Torus palatinus can lead to loss of teeth or malocclusion. We present the case of an 18-year-old female patient found to have a heterozygous variant of the LRP5 mutation on genetic testing. Given the rarity of this disease, long-term sequelae and treatment options are not fully understood.

## Introduction

Mutations in the low-density lipoprotein receptor-related protein 5 (LRP5) gene, located on human chromosome 11q13.4, can lead to Worth syndrome [1,2]. Pathogenic variants in this gene are involved in the canonical Wnt pathway [2-5]. Loss-of-function variants can cause osteoporosis-pseudoglioma syndrome, while gain-of-function variants result in a high bone mass phenotype, referred to as high bone mineral density LRP5 (LRP5 HBM) [5].

LRP5 HBM leads to diffuse skeletal densification, particularly of the tubular long bones and cranial vault; however, these skeletal changes do not seem to lead to an increased risk of fracture [6]. This syndrome was first recognized in 1966 by H.M. Worth [3,7]. In 1977, L. Glass and R.J. Gorlin differentiated the syndrome from van Buchem disease (hyperostosis corticalis generalisata) by the dominant inheritance pattern and absence of hypertelorism, exophthalmos, nasal obstruction, increased head circumference, cranial nerve involvement, and elevated alkaline phosphatase [3,7]. In 1987, a group of physicians from Spain suggested that the syndrome may sometimes cause nerve damage and that it may not be completely benign as previously suggested [3,7]. 

Based on a historical review published in September 2023, 155 cases had been reported to date of the autosomal dominant variant of Worth disease [1]. Heterozygous variants have also since been identified although even more rarely reported. Here, we present the case of a young female patient diagnosed with a heterozygous variant of the LRP5 gene (c.844A>G, p.Met282Val) leading to hyperostosis to explore possible complications and treatment options for this disease. 

## Case presentation

**Figure 1:**
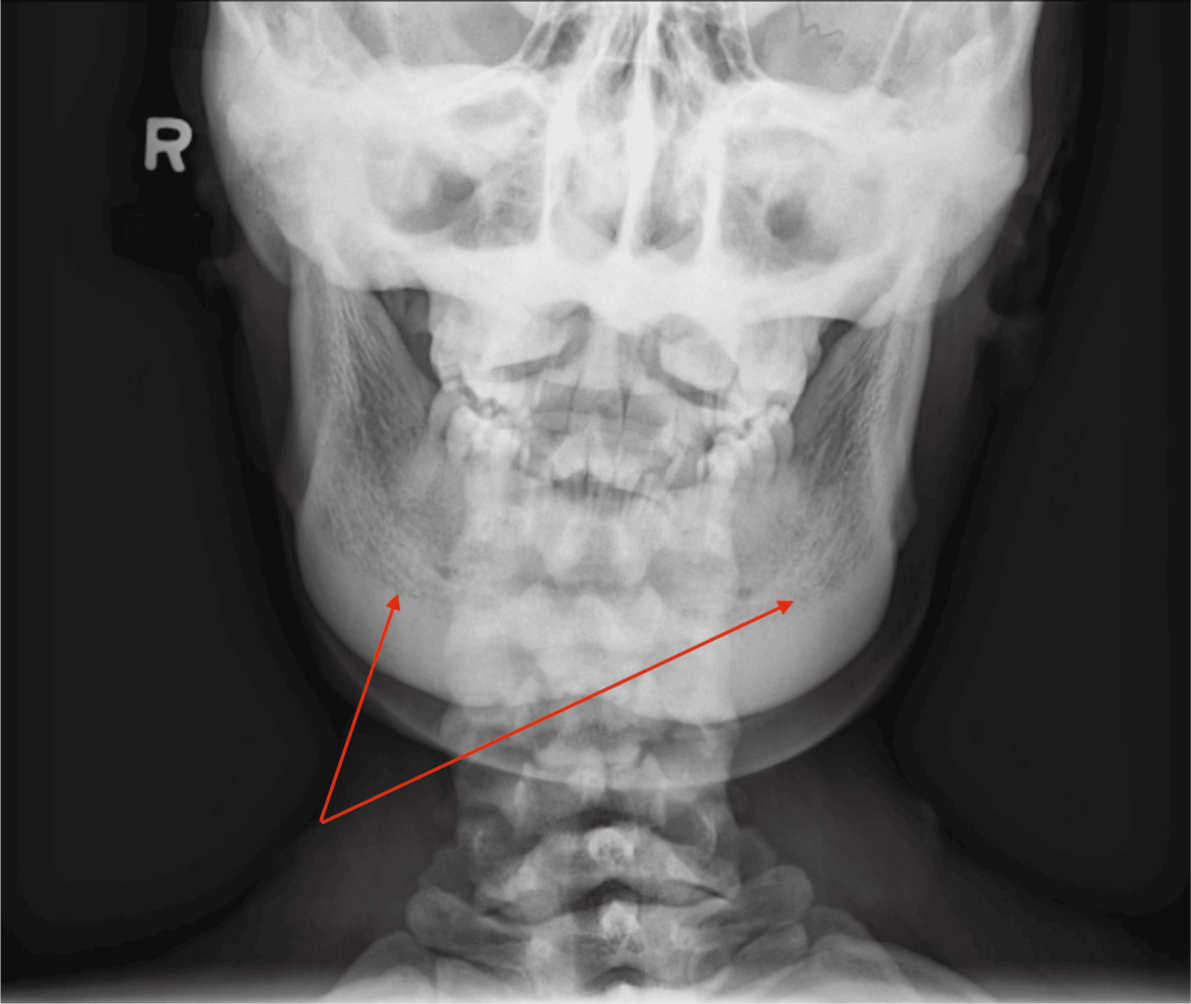
Posteroanterior X-ray of the mandible Diffusely increased bone mineral density with thickening of bilateral mandibular bodies

**Figure 2:**
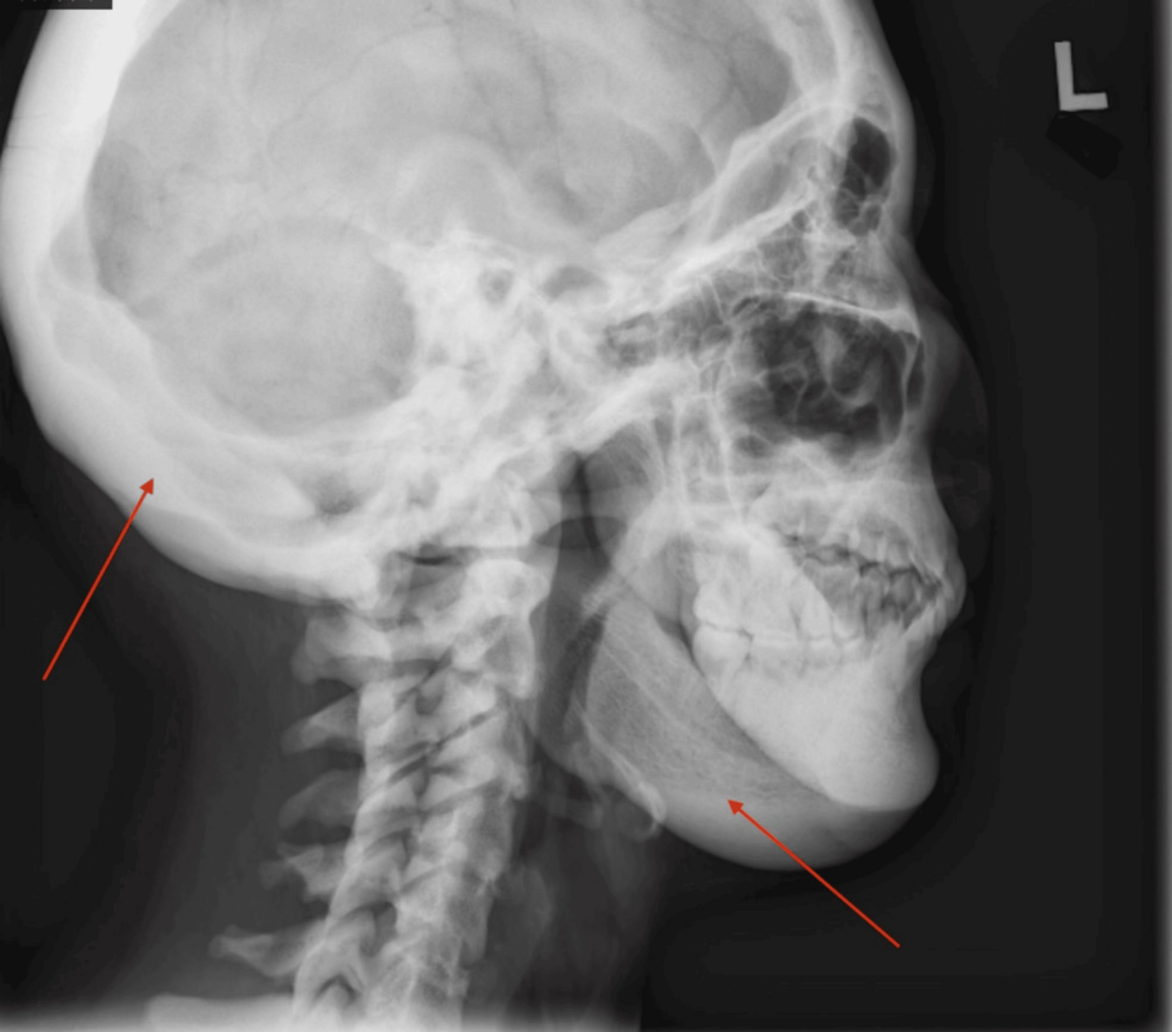
Lateral left X-ray of the mandible Increased bone mineral density of the mandibular body and symphysis and the skull

An 18-year-old female patient with a history of high bone mass was referred to Genetics for evaluation. Concern for the patient's health began with an observation made by her dentist during a routine dental X-ray where a highly mineralized bone in the jaw was noted (Figure 1 and Figure 2). The only complaint that the patient had at that time was minimal facial discomfort while noticing some broadening of her jaw. There is no family history of osteoporosis, fractures, or increased bone mineral density. On initial presentation, her head circumference was above 2 standard deviations from the mean. Otherwise, her growth and development was normal. Notable portions of the physical exam included full cheeks with a squared-off jaw, particularly at the mandibular rami. She has torus palatinus with normal teeth and gums. A skeletal survey demonstrated "diffusely increased bony mineralization and cortical thickening throughout the axial and appendicular skeleton, but without the characteristic bone-within-bone age characteristic of osteopetrosis." A CT scan of the mandible showed "diffusely increased bone mineral density with thickening of the bilateral mandibular body and symphysis." Dual-energy X-ray absorptiometry (DEXA) showed a bone mineral density of 1.973 g/cm^2^ in the L1-L4 spine (Z-score = +6.3) and 2.088 g/cm^2^ in the left total hip (Z-score = +8.6). A salivary Skeletal Dysplasias Core Panel testing for 358 genes revealed the patient has a de novo variant in the heterozygous state LRP5 (c.844A>G, p.Met282Val-uncertain significance). She was referred to Ophthalmic Genetics who found a normal eye exam. She continues to have a close follow-up with her dentist.

## Discussion

Our case represents a de novo heterozygous variant of the LRP5 gene. Patients harboring LRP5 HBM variants share a similar skeletal picture with a predominant involvement of the skull and cortices of the long bones [8]. Based on literature review, hyperostosis has been reported in one patient with the same variant as our patient [9]. The M282V variant was seen in a 55-year-old woman in which hyperostosis was found incidentally at the age of 37. The physical exam showed normal height and weight, but a torus palatinus was notable. Radiographic studies of several skeletal sites showed thickening of the skull and long bones. At ages 50 and 55, she had DEXA scans, the most recent of which revealed that the bone mineral density at the left total hip, femoral neck, and L1-L4 spine were 1.862 g/cm^2^ (Z-score = +8.23), 1.638 g/cm^2^ (Z-score = +8.18), and 1.613 g/cm^2^ (Z-score = +6.23), respectively [9]. 

The LRP5 HBM mutations are all located in the first YWTD β-propeller domain of LRP5 [9]. A homology model for this domain showed that the M282V variant is adjacent to other high bone mineral density mutations [9]. Clinical manifestations of these mutations can vary but frequently include torus palatinus, a wide and deep mandible, and less commonly neurological complications such as optic nerve compression and hearing loss [1,9,10]. 

There is no reported treatment directed against this specific mutation or LRP5 HBM in general. There are cancer treatments (experimental at this time) that inhibit Wnt signaling and may be a possibility in the future, particularly porcupine inhibitors which interfere with Wnt ligands [3,11]. In vitro studies also show a potential treatment with a signaling protein Dickkopf-1 (Dkk-1), which has been shown to be involved in the normal inhibition of Wnt signaling [5]. There is a potential treatment in women carrying this mutation with depot medroxyprogesterone acetate (DMPA), an injectable contraceptive administered four times per year [12]. Its use leads to loss of bone mineral density; however, current evidence shows the recovery of bone density when DMPA is discontinued [12]. The decrease in bone mineral density with DMPA is about 5% and was deemed to be not clinically relevant for our asymptomatic patient [12]. In our case, we opted to continue observation with routine dental, audiology, and ophthalmology follow-up. Lastly, we discussed that there could also be a role for treatment with steroids, which we do not recommend due to the secondary effects of long-term use. Finally, surgical interventions can also be considered if localized facial deformities or entrapment neuropathies result. 

## Conclusions

We present a case of a heterozygous variant of Worth-type endosteal hyperostosis, more recently referred to as LRP5 HBM. Neurologic complications, which were previously thought to be absent, may affect a small portion of patients with the autosomal dominant variant. These manifest largely as entrapment neuropathies such as hearing loss and rarely as circulation problems in the brain. Such findings have not been reported in heterozygous cases, although these were much less found in the literature. When a case of hyperostosis is suspected, genetic analysis leading to the appropriate diagnosis of LRP5 HBM is fundamental to continue to better our understanding of this disease.

The LRP5 HBM mutation increases Wnt signaling by impairing the action of an antagonist of the Wnt pathway. These findings point to the role of altered LRP5 function leading to high bone mineral density and may point at potential targets for the prevention or treatment of this disease. 
